# Screening for retinopathy of prematurity in South Africa: are those developing severe ROP screened on time? Data from a prospective register

**DOI:** 10.1136/bmjophth-2025-002239

**Published:** 2025-07-13

**Authors:** Tshilidzi van der Lecq, Natasha Rhoda, Esmè Jordaan, Nicola Freeman, Lloyd Tooke, Rudzani Muloiwa, Clare Gilbert, Gerd Holmstrom, on behalf of the ROPSA Collaborative Group, Tshilidzi van der Lecq

**Affiliations:** 1 Department of Surgery, Division of Ophthalmology, University of Cape Town, Cape Town, South Africa; 2 Department of Paediatrics & Child Health, University of Cape Town, Cape Town, South Africa; 3 Biostatistics Research Unit, South African Medical Research Council, Cape Town, South Africa; 4 Statistics and Population Studies Department, University of the Western Cape, Cape Town, South Africa; 5 Clinical Research, London School of Hygiene and Tropical Medicine, London, UK; 6 Department of Surgical Sciences, Ophthalmology, Uppsala University, Uppsala, Sweden

**Keywords:** Child health (paediatrics), Retina, Epidemiology

## Abstract

**Background/Aims:**

To determine whether retinopathy of prematurity (ROP) screening is initiated on time according to current South African (SA) guidelines, that is, before the onset of stage 3 and type 1 ROP.

**Methods:**

A prospective study of preterm infants screened at five neonatal units between 1 May 2022 and 31 January 2023 in Cape Town, SA. Data on all infants screened with a birth weight <1250 g or gestational age (GA) <32 weeks were extracted from the ROP South African (ROPSA) register, including postnatal age (PNA) and postmenstrual age (PMA) at first screening.

**Results:**

A total of 696 infants were included, 58.9% (n=410) of whom had an early ultrasound (EUS) for GA estimation. Overall, 220 (31.6%) infants developed ROP, 20 (2.9%) had stage 3 or type 1 and 7 (1.0%) required treatment. Screening was initiated on time according to SA criteria in 549 (78.9%) infants, none of whom had stage 3 or type 1 ROP at first screening. Stage 3 and type 1 ROP were first detected at PNA and PMA of 6.3 and 33.1 and 8.9 and 35.9 weeks, respectively. Most infants (319, 45.8%) were screened according to PNA only, and 78.9% of the 185 infants screened only once did not attend subsequent examinations.

**Conclusion:**

Screening started on time in most infants and prior to the development of severe ROP. Due to the limited availability of EUS in our region and to promote complete screening, we recommend that screening be initiated using PNA alone at 4–6 weeks or prior to discharge, whichever is earliest. The low proportion of infants with stage 3 and type 1 ROP is a limitation in our study. Therefore, recommendations may not be generalisable to South African regions where neonatal care results in a higher proportion of infants developing type 1 ROP.

**WHAT IS ALREADY KNOWN ON THIS TOPIC**

- Improvement in the quality of neonatal care in low- and middle-income countries (LMICs) is leading to a greater survival of preterm infants, including extremely low birth weight (birth weight <1000 g) infants who are most at risk of severe retinopathy of prematurity (ROP). In these settings, infants at risk of requiring treatment for ROP (ie, type 1 ROP) differ from those in high-income countries. Even in LMICs with established national ROP screening guidelines, there is little up-to-date evidence on when screening should start for the timely detection of type 1 ROP.

**WHAT THIS STUDY ADDS**

- In our setting, most infants are screened on time according to the current guidelines. Stage 3 or type 1 ROP was only diagnosed at delayed first examination in three infants. There is limited availability of early ultrasound which may affect the accuracy of screening initiation using postmenstrual age (gestational age plus postnatal age (PNA) in weeks). We recommend that screening be initiated using PNA only, at 4–6 weeks or prior to discharge (whichever comes earliest).

**HOW THIS STUDY MIGHT AFFECT RESEARCH, PRACTICE OR POLICY**

- These findings will contribute to the criteria used to initiate ROP screening in SA as the national guidelines published in 2012 are currently under review. Further studies are required outside Cape Town where the quality of neonatal care is likely to differ, influencing the onset of severe ROP. Scaling up the ROP South African (ROPSA) register to other regions of the country would provide comparable data to assess the adequacy of these criteria in the future.

## Introduction

 Retinopathy of prematurity (ROP) is an important cause of blindness in children. Evidence suggests that the third epidemic of ROP blindness is currently affecting low- and middle-income countries (LMICs), including those in Sub-Saharan Africa (SSA).^1^,^3^ In Africa in the late 1990s, ROP blindness was only reported in South Africa (SA) and Nigeria,^4^ but ROP blind infants have recently been reported from other African countries.^3^ A 2020 model estimated that 65% of preterm births occurred in Asia and SSA.^5^ Policies and programmes to reduce neonatal mortality rates in response to the United Nations Sustainable Development Goals are increasing the survival of infants at risk of ROP in SSA countries including SA.^6 7^

ROP screening programmes are a cost-effective way of reducing blindness in preterm infants, ensuring that treatment requiring ROP (type 1 ROP) is detected and treated in a timely manner.^8^ South Africa’s national ROP guidelines for the prevention, screening and treatment of ROP helped to establish ROP screening programmes throughout the country. These guidelines recommend screening infants with a birth weight (BW) of <1500 g, or a gestational age (GA) of <32 completed weeks, or a BW between 1500 g and 2000 g if there are additional risk factors. In addition, the guidelines recommend that screening starts at a postnatal age (PNA) of 4–6 weeks or a postmenstrual age (PMA, GA plus PNA) of 31–33 weeks whichever comes later.^9^ No study has assessed whether ROP screening is currently initiated based on these criteria, nor whether the recommendations are suitable for the population of infants at risk in SA.

The effectiveness of ROP screening programmes requires an understanding of the age of onset of ROP in a given population.^10^ However, most studies have been undertaken in high-income countries (HICs) where infants most at risk of severe ROP have a BW of <1000 g or a GA of <30 weeks. Guidelines arising from these studies recommend the use of PMA to guide the timing of the first examination.^11^,^15^ The criteria currently used in SA have modified these recommendations without the benefit of population-specific data.

Early ultrasound (EUS) provides the highest level of certainty in estimating GA, while postnatal assessments using the New Ballard Score or foot length provide the lowest level of certainty.^16 17^ However, in LMICs, EUS is not readily available for most pregnancies.^18^ In this context, postnatal assessments can overestimate or underestimate GA.^19 20^ For example, in a South African study that predates the SA guidelines, early ultrasound was used to estimate GA in only 10% of 145 infants screened for ROP.^21^ Inaccurate GA estimates may lead to suboptimal timing of the first screening examination and a delay in the detection of treatment-requiring ROP.

The aim of this study is to determine whether ROP screening starts on time according to the current South African guidelines, and whether the PNA and PMA recommendations for first screening are adequate given the age of onset of severe ROP (stage 3 or type 1 ROP) in this region.

## Subjects and Methods

### Study population

This prospective cohort study included infants sequentially identified and screened for ROP in five public sector units located in the Cape Town Metropole region of the Western Cape Province, South Africa. These five units provide ROP screening for 75% of the regional population (4.7 million).^22 23^ The regional criteria for ROP screening are a GA of <32 completed weeks or a BW of <1250 g. Infants with a GA≥32 weeks or BW≥1250 g with an unstable clinical course may also be screened as recommended by paediatricians.^9^ It is important to note that a lower BW (<1250 g) criterion is used in the Cape Town Metropole region compared with the <1500 g recommended in the SA guidelines. The lower BW criterion used is in keeping with an evidence-based 2020 regional policy decision which considered the resources available for ROP screening and the number of infants requiring screening. All other aspects of the South African national guidelines are adhered to in the region.

Preterm infants born between 1 May 2022 and 31 January 2023, who received intensive neonatal care and survived until the time of first screening examination (ie, 6 weeks PNA or 33 weeks PMA) were included. EUS was available for GA estimation in 410 (58.9%) infants. In other infants, GA was estimated by postnatal foot length in 153 (22.0%), New Ballard score in 65 (9.3%), late ultrasound in 36 (5.2%) or was undocumented in 32 (4.6%).

### Screening procedure

Infants were eligible for screening based on the regional criteria (GA<32 weeks or BW<1250 g). The timing and logistics of screening for ROP were conducted according to the South African guidelines.^9^ The first screening examination was scheduled for 4–6 weeks PNA or a PMA of 31–33 weeks, whichever came later. Screening was conducted weekly or two times per week by ophthalmologists or supervised ophthalmology residents. Infants who met the South African criteria for the termination of screening were considered to have completed the screening process. These included infants attaining (a) 45 weeks PMA with no prethreshold disease, or (b) zone 3 vascularisation without previous zone 1 or 2 ROP, or (c) complete regression of ROP, or (d) full retinal vascularisation. Infants not meeting these criteria were regarded as having incomplete screening.

Retinal findings were documented on standardised ROP templates, as suggested in the South African guidelines. Signs of ROP were classified using the revised International Classification of ROP (ICROP 3).^24^ Infants with type 1 ROP (ie, any stage ROP in zone 1 with plus disease, stage 3 in zone 1 without plus disease; or stage 2 or 3 ROP with plus disease in zone 2) in either eye were referred for treatment.^25^

### Data management

Data on the cohort of infants were extracted from the electronic ROP South African (ROPSA) register.^26^ This register collects data on infants screened for ROP in five public sector neonatal units in the Cape Town Metropole region in SA. These infants were either born at these units or referred from other units. The register collects neonatal, ophthalmic and treatment data related to ROP. Patients or the public were not involved in the design, or conduct, or reporting, or dissemination plans of our research.

### Statistical analysis

Neonatal characteristics, BW in grams, GA in weeks as well as the PNA and PMA were considered normally distributed and were described using means, SD and ranges. T-tests were performed to compare groups of infants regarding their mean GA and BW, and mean PNA and PMA at first examination.

Screening initiated at or before 6 weeks PNA or 33 weeks PMA was considered to be performed on time. Descriptive statistics were used for the number (n, %) of infants who were screened on time as well as the number of infants who presented with each stage of ROP. Associations between categorical variables were established using χ^2^ tests. All analyses were performed using SAS V.9.4 statistical software (SAS Institute, Cary, North Carolina, USA).

## Results

A total of 696 infants who met the screening criteria were included; 355 (51.0%) were female and 548 (79.7%) were singleton births. The infants had a mean GA of 28.9 (SD 1.7, range 24–36) weeks and a mean BW of 1080.9 (SD 218.4, range 640–1840) grams. 274 (39.4%) infants had a BW<1000 g (extremely low birth weight, ELBW) and 181 (26.0%) had a GA<28 weeks. Most infants, 378 (54.3%) completed the screening process, 318 (45.4%) did not. ROP developed in 220 (31.6%, 95% CI 28.3 to 35.3) infants (table 1), 7 of whom required treatment.^26^

**Table 1:** Characteristics of all screened infants (n=696) and of those with ROP (n=220)

		All infants (n=696)		Infants with any ROP (n=220)		P value*
		n	%	n	%	
Sex	Male	341	49.0	113	51.4	
	Female	355	51.0	107	48.6	0.395
Gestational age	<28 weeks	181	26.0	72	32.7	
	≥28 weeks	515	74.0	148	67.3	0.007
Birth weight	<1000 g	274	39.4	118	53.6	
	≥1000 g	422	60.6	102	46.4	<0.001
Gestational age estimation method†	Early ultrasound	410	61.8	120	54.5	
	Other methods	254	38.2	105	47.7	<0.001

* P value for difference between infants with ROP and infants with no ROP.

† Data missing for 32 infants.

ROP, retinopathy of prematurity.

EUS was used to estimate GA in 100 (58.1%) infants with GA<28 weeks and 310 (63.0%) infants with GA≥28 weeks (p=0.260). In infants with a BW<1000 g, EUS was used for 179 (68.9%) infants compared with 231 (57.1%) infants with BW≥1000 g (p=0.002).

### Timing of first screening examination

Overall, the first screening examination occurred at a mean PNA of 5.3 (SD 2.0, range 1.3–16.7) weeks and a mean PMA of 34 (SD 2.3, range 27.7–47) weeks. According to PNA, 534 (76.7%) infants had their first examination at ≤6 weeks, 424 (60.9%) infants at ≤5 weeks and 143 (20.5%) infants at ≤4 weeks (online supplemental figure 1). Among the 185 infants who were screened only once, most (146, 78.0%) failed to attend subsequent examinations.^26^

The first screening examination was performed on time in 549 (78.9%) infants according to PNA and/or PMA criteria.

The ELBW infants were screened at a later PNA than infants with a BW≥1000 g (p=0.009) and at an earlier PMA (33.7 (SD 2.1, range 28.3–44.4)) weeks and 34.6 (SD 2.4, range 27.7–47.0), respectively) (p<0.001) (table 2).

**Table 2:** Criteria fulfilled for first screening, by birth weight

Birth weight	PNA criterion only		PMA criterion only		PNA and PMA criteria		Later than both criteria		Mean PNA at screening weeks (SD, range)
	N	%	N	%	N	%	N	%	
<1000 g (274)	89	32.5	14	5.1	107	39.1	64	23.4	5.6 (2.0, 51.7–14.1)
≥1000 g (422)	230	54.5	1	0.2	108	25.6	83	19.7	5.1 (2.0, 1.3–16.7)
Total (696)	319	45.8	15	2.2	215	30.9	147	21.1	5.3 (2.0, 1.3–16.7)

PMA, postmenstrual age; PNA, postnatal age.

### Timing of first detection of ROP

**Figure 1:**
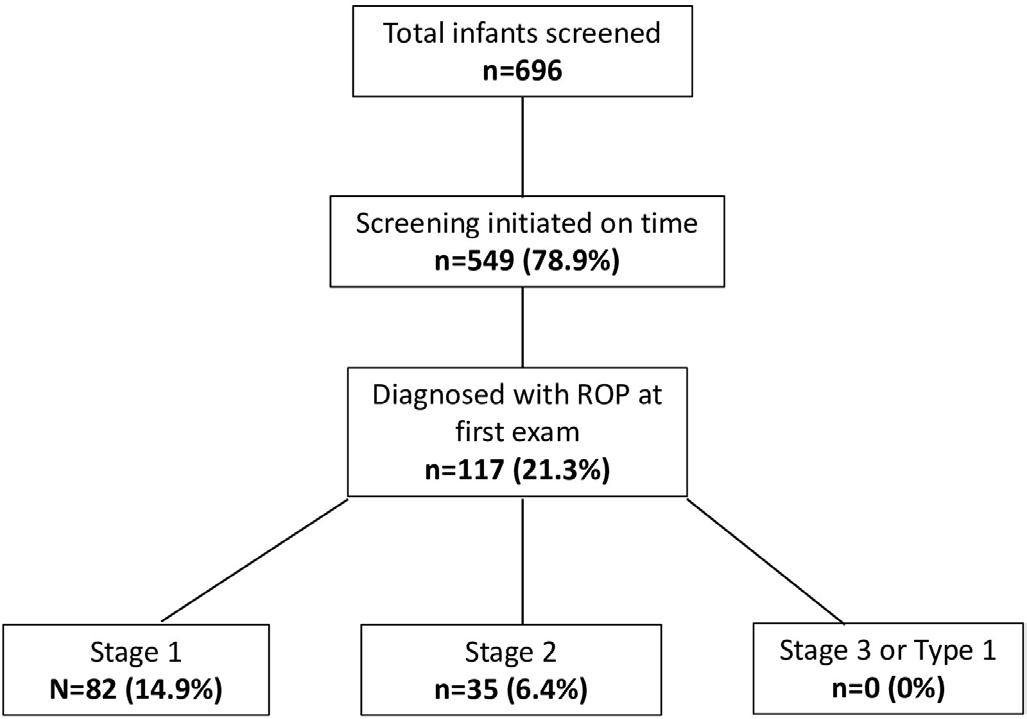
Stage of retinopathy of prematurity (ROP) in infants screened on time.

Among the 549 infants screened on time, 117 (21.3%) were diagnosed with ROP. None of these infants had stage 3 or type 1 ROP at this first examination (figure 1).

Among all 696 infants, ROP was diagnosed at the first screening in 152/696 (21.8%) infants, representing 69.1% (152/220) of those who developed ROP. The mean PNA and PMA at their first examination were 5.5 (SD 1.7, range 1.7–11.4) and 34.1 (SD 1.9, range 27.7–41.1) weeks, respectively.

In the 544 infants with no ROP at the first screening, another 68 (30.9%, 68/220) infants went on to develop some stage of ROP in one or both eyes. In these 68 infants, the mean PNA and PMA at detection of ROP were 8.8 (SD 2.9, range 3.9–21.3) weeks and 37.1 (SD 2.9, range 33–50.0) weeks, respectively.

### Timing of first detection of ROP stage 3 and type 1 ROP

Among the 220 infants with ROP, the most advanced stage of ROP detected was stage 1 in 134 (60.9%), stage 2 in 67 (30.5%) and stage 3 in 19 (8.6%) in at least one eye. No infant developed stage 4 or 5 ROP.

Among the 19 infants who developed stage 3 ROP in at least one eye, three were diagnosed at delayed first screening examination, which took place at PNA and PMAs of 7 and 33.1 weeks, 7.6 and 36.6 weeks, and 9.7 and 37.3 weeks, respectively. 16 infants developed stage 3 ROP in either eye after the first examination at a mean PNA of 11.8 (range 6.3–21.9) weeks and a mean PMA of 38.2 (range 32.9–49.9) weeks (table 3).

**Table 3:** Characteristics of infants with ROP stage 3 and/or type 1 ROP

Infant	BW (grams)	GA (weeks)	Sex	PMA (weeks) at onset of stage 3	PNA (weeks) at onset of stage 3	PMA (weeks) at onset oftype 1 ROP	PNA (weeks) at onset oftype 1 ROP
Stage 3 at first screening							
1*	875	26	F	33.1	7	39.1	13.0
2*	880	29	F	36.6	7.6	36.6	7.6
3	975	27	M	37.3	9.7	–	–
No ROP at first examination; developed stage 3 later							
4*	1050	29	F	37.7	8.7	38.3	9.3
5	800	28	F	37	9	–	–
6	1080	29	M	40	10.9	–	–
7	1280	29	M	36.1	7.1	–	–
8	1310	29	F	35.3	6.3	–	–
ROP at first examination which progressed to stage 3 later							
9*	725	28	F	35.4	6.9	37.4	8.9
10*	865	25	F	35.7	10.6	36.3	10.6
11*	910	27	F	34.9	7.9	35.9	8.9
12	785	30	F	45.3	15.4	–	–
13	815	25	F	36.6	7.5	–	–
14	865	26	M	32.9	6.9	–	–
15	875	27	F	33.6	6.6	–	–
16	895	28	F	44	15	–	–
17	1040	28	M	37.7	9.7	–	–
18*†	1065	26	F	–	–	37.0	11.6
19	1070	28	M	39.3	11.3	–	–
20	1170	29	F	39	10	–	–

* Infants who developed type 1 ROP.

† Infant who developed type 1 ROP, but a maximum stage 2 ROP.

BW, birth weight; GA, gestational age; PMA, postmenstrual age; PNA, postnatal age; ROP, retinopathy of prematurity.

Seven (1%) infants developed type 1 ROP and all were treated. One of these infants was diagnosed at a delayed first screening at a PNA of 7.6 weeks and a PMA of 36.6 weeks. In the remaining six infants, type 1 ROP was detected at a mean PNA of 10.1 (range 8.9–13.0) weeks and a mean PMA of 37.4 (range 35.9–39.1) (table 3).

Five of the seven (71.4%) treated infants were ELBW. Among the seven treated infants, six received an anti-vascular endothelial growth factor (anti-VEGF) injection (Avastin) as primary treatment in both eyes. Only one infant received laser in the right eye (due to the presence of early retinal traction) and an anti-VEGF injection in the left eye. All infants were treated within 72 hours of the diagnosis of type 1 ROP.

## Discussion

In our study in a metropolitan region of SA, screening was initiated on time in most (549, 78.9%) infants, according to PNA and/or PMA criteria, and no infant screened on time was diagnosed with type 1 ROP at the first screening examination.

The current guidelines in SA recommend that screening should start using PNA or PMA, whichever is the latest. Initiation of screening should, therefore, be based on PNA in infants with a GA of ≥28 weeks and delayed to a PMA≥34 weeks. In extremely preterm (<28 weeks or BW<1000 g) infants, screening should start using PMA, delayed to a PMA of 33 weeks (online supplemental table 1).

**Figure 2:**
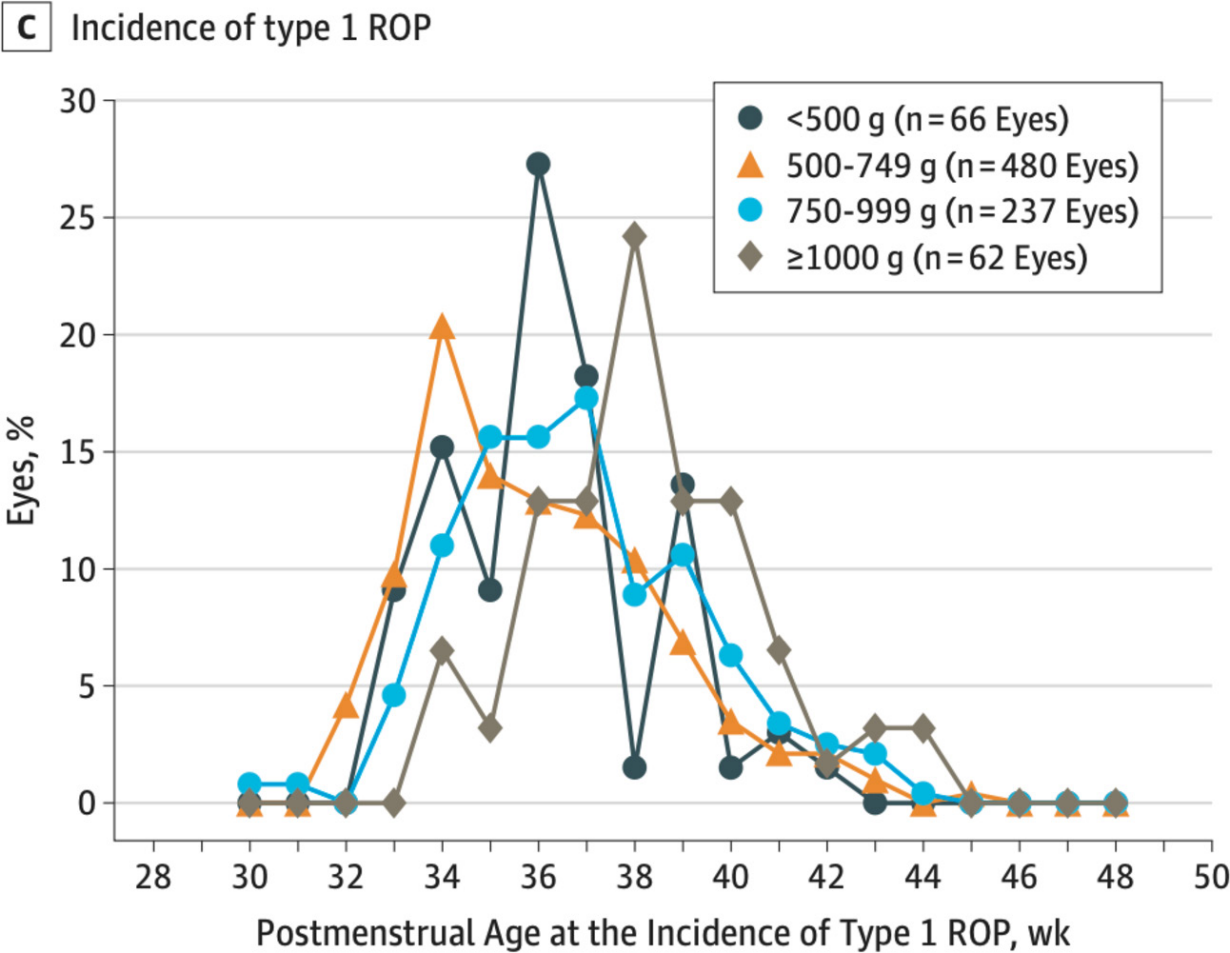
Postmenstrual age at onset of type 1 ROP for different birth weight categories^15^ (^*^with permission from *JAMA Ophthalmology* and the first author). ROP, retinopathy of prematurity. *Reproduced with permission from *JAMA Ophthalmology*, 2018, 136(12): 1383–1389. Copyright (2018) American Medical Association. All rights reserved, including those for text and data mining, AI training and similar technologies.

A large study from the USA showed that the distribution curves of the incidence of type 1 ROP in different GA groups shift to the right with increasing GA (figure 2). In this study, 11% of eyes in ELBW infants had developed type 1 ROP by a PMA of 33 weeks.^15^ In our study, the ‘later’ PMA criterion was used to initiate screening in only 15 of the 274 ELBW infants. Although no infant developed type 1 ROP at a PMA of ≤33 weeks, three infants (GA 26, 26 and 27 weeks) developed stage 3 ROP at PMAs of 32.9, 33.1 and 33.6 weeks.

Prioritising the later PMA criterion in ELBW infants may result in a later PNA of first screening for infants with GAs of 26, 27 and 28 weeks, which corresponds to PNAs of 7, 8 and 9 weeks, respectively. Interestingly, a systematic review of screening guidelines showed that the current guidelines in SA on when to start screening infants with GA<28 weeks are at a later PNA than other HIC and LMICs.^27^

An accurate PMA relies on an accurate method of GA estimation. In our study, EUS was available for just over half of the screened infants. This may be an overestimation for the region, as regional data from a pregnancy register of 14 527 women found that only 23% (n=3345) had undergone EUS.^28^ This reflects the limited availability of EUS in SA, which is also likely in other less well-resourced countries.^18^ The reliance on less accurate methods of GA estimation,^19^ suggests that initiating screening based on PNA alone is more reliable in this setting and would be easier for neonatal staff and parents to understand and remember. Initiating screening based on a consistent PNA criterion may also reduce unnecessary early examinations performed at ≤4 weeks PNA in 143 (20.5%) infants in our study, which would increase the cost-effectiveness of screening.

While the first screening was delayed in approximately 20% of the infants, almost 80% (146/185) of those screened only once did not return for further examinations. Initiating screening prior to discharge has been shown to increase the proportion of eligible infants who are screened, and if accompanied by the counselling of parents, increases the proportion of infants who complete screening.^29^ This approach could increase the number of infants with screening initiated on time and reduce non-adherence to follow-up screening.

A limitation of our study is the low proportion of infants developing type 1 ROP (1%) despite the large number of infants screened, which limits the generalisability of the findings. There are several possible explanations for the low rate of type 1 ROP. First, some of the infants who failed to attend follow-up screening may have subsequently developed type 1 ROP, and second, in our setting, infants with a GA<24 weeks and BW<500 g are not actively managed or routinely screened due to their high mortality rate. There is also evidence to suggest that African heritage may be associated with a lower risk of type 1 ROP.^30^ However, data on ethnicity are not routinely collected in SA.

Based on the findings of this study, we recommend that screening should start according to a PNA criterion alone at 4–6 weeks or prior to discharge (whichever comes first). This would avoid less reliable and delayed first screening based on PMA, particularly for ELBW infants without early ultrasound. To determine whether our recommendations are applicable in a more representative sample in SA and in other regions using the national BW criterion, the current ROPSA register will need to be adapted and scaled up countrywide.

## Conclusion

This study, which used data from the ROPSA register, demonstrates the benefit of monitoring when screening starts and the onset of ROP in established ROP screening programmes in LMICs. As improvements in neonatal care increase the survival of extremely preterm infants, screening guidelines may need to be adjusted to ensure the timely detection of type 1 ROP. Continuous surveillance facilitated by the ROPSA register will be helpful to ensure the timely diagnosis of treatment requiring ROP in these vulnerable infants.

## Supplementary material

10.1136/bmjophth-2025-002239 online supplemental figure 1

10.1136/bmjophth-2025-002239 online supplemental table 1

## Data Availability

Data are available upon reasonable request.
