# Supplementary material for: Screening for retinopathy of prematurity in South Africa: are those developing severe ROP screened on time? Data from a prospective register
Source: BMJ Open Ophthalmol. 2025 Jul 13;10(1):e002239. doi: 10.1136/bmjophth-2025-002239 (PMC12258271; doi:10.1136/bmjophth-2025-002239)
Supplement: online supplemental figure 1 [file bmjophth-10-1-s001.pdf]

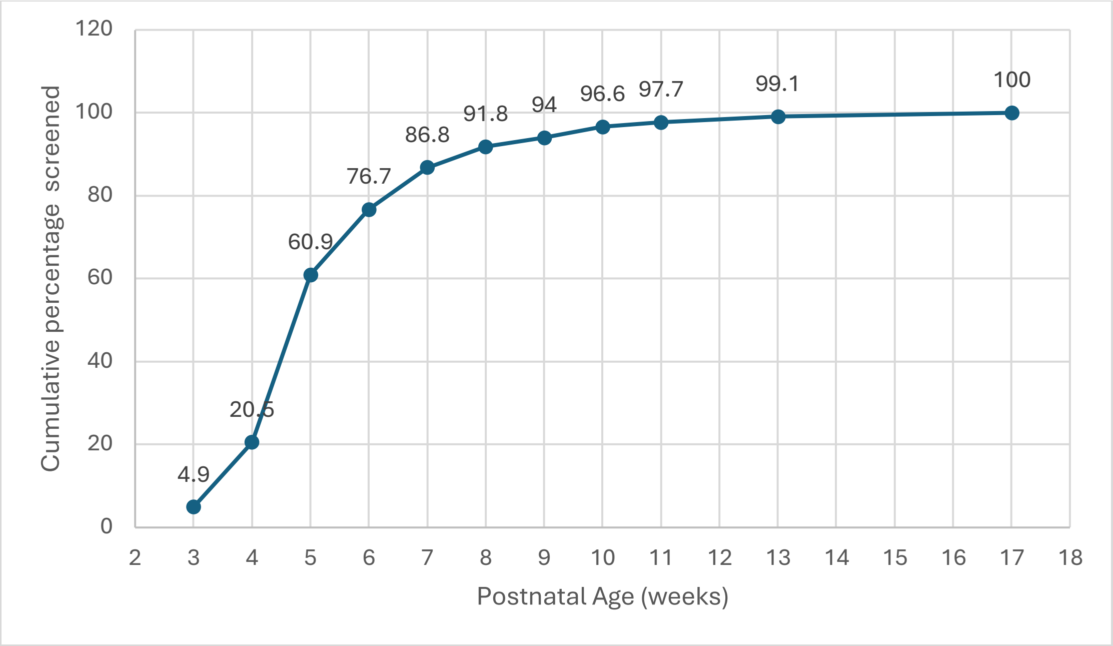

Note: values from 3 week's PNA onwards include examinations performed prior to 3 weeks PNA

Supplemental Figure 1. Timing of first screening examination by postnatal age (n=696)
