# Supplementary material for: Screening for retinopathy of prematurity in South Africa: are those developing severe ROP screened on time? Data from a prospective register
Source: BMJ Open Ophthalmol. 2025 Jul 13;10(1):e002239. doi: 10.1136/bmjophth-2025-002239 (PMC12258271; doi:10.1136/bmjophth-2025-002239)
Supplement: online supplemental table 1 [file bmjophth-10-1-s002.pdf]

Supplemental Table 1. Comparison of criteria used to initiate screening according to gestational age

Gestational age	PNA of 6 weeks (PMA = GA +6)	PMA of 33 weeks (PNA = PMA-GA)
24	6 (30)	**33 (9)**
25	6 (31)	**33 (8)**
26	6 (32)	**33 (7)**
27	**6 (33)**	**33 (6)**
28	**6 (34)**	33
29	**6 (35)**	33
30	**6 (36)**	33
31	**6 (37)**	33
32	**6 (38)**	33

Bold font is the recommended ‘later’ criterion for screening initiation
